# Research Progress of Nerve Injury–Induced Protein 1 in Cardiovascular Diseases

**DOI:** 10.1155/crp/9683634

**Published:** 2026-04-15

**Authors:** Xiaohui Xu, Yuming Mu

**Affiliations:** ^1^ Department of Echocardiography, The First Affiliated Hospital of Xinjiang Medical University, 137 Liyushan South Road Xinshi District, Urumqi, 830000, Xinjiang, China, xjmu.edu.cn; ^2^ Xinjiang Key Laboratory of Ultrasound Medicine, 137 Liyushan South Road Xinshi District, Urumqi, 830000, Xinjiang, China

**Keywords:** cardiovascular diseases, Ninj1

## Abstract

Nerve injury–induced protein 1 (Ninjurin 1) is a cell surface adhesion molecule that contains one extracellular adhesion domain and two transmembrane domains. Originally discovered in nerve injury, it has been extensively studied for its role in nerve regeneration. Ninj1 mediates the transendothelial migration of myeloid cells mainly through the extracellular adhesion domain, thereby aggravating the inflammation of the central nervous system. In addition to regulating the inflammatory phenotype of cells, Ninj1 actively mediates the rupture of the plasma membrane and regulates the programmed death of inflammatory cells, thereby participating in the host defense against exogenous infection. In recent years, Ninj1, an important protein associated with inflammasome activation, has garnered increasing attention from researchers regarding its mechanistic role and pathophysiological significance in cardiovascular diseases. Accumulating evidence suggests that Ninj1 not only participates in the regulation of inflammatory responses and cell death processes but also plays a critical role in the onset and progression of various cardiovascular conditions, including atherosclerosis, myocardial ischemia–reperfusion injury, and heart failure. Therefore, an in‐depth exploration of the specific functions of Ninj1 in the cardiovascular system holds significant scientific and clinical value for elucidating the molecular mechanisms underlying these diseases and for developing novel therapeutic strategies. This review aims to summarize the research progress on Ninj1 in cardiovascular diseases and to outline its mechanisms in pathological processes.

## 1. Introduction

Cardiovascular diseases (CVDs) are the leading cause of death worldwide and pose a serious threat to human health. CVD has become the leading cause of death in China and a major public health problem. The pathogenesis of common CVDs, such as atherosclerosis (AS) and cardiomyopathy, is complex, involving multiple pathological processes, such as inflammatory response, oxidative stress, apoptosis, and vascular endothelial dysfunction. With the deepening of research, more and more molecular targets have been found to be closely related to the occurrence and development of CVDs. Nerve injury–induced protein 1 (Ninjurin 1), or Ninj1, is an adhesion molecule first discovered in Schwann cells after nerve injury. It is induced under inflammatory conditions and is a nerve injury–induced two‐channel membrane protein. It is also a potential adhesion molecule. Accumulating evidence suggests that Ninj1 mediates cell communication; is required for leukocyte entry, adhesion, activation, and motility during tissue remodeling; and may be a key regulator in regulating leukocyte adhesion and trafficking in inflammation and leukocyte‐mediated diseases. Moreover, recent studies have shown that Ninj1 is involved in a variety of physiological processes, such as nerve regeneration, inflammation, and tissue homeostasis [1–4]. The expression and function of Ninj1 exhibit diversity across multiple cell types, including endothelial cells (ECs), smooth muscle cells, and immune cells, all of which play critical roles in the development of CVD. Moreover, by regulating various signaling pathways, Ninj1 exerts key physiological functions during the progression of CVD [5, 6]. Of particular note, Ninj1 serves as a critical regulator of plasma membrane rupture (PMR) during inflammatory cell death, thereby amplifying the inflammatory cascade under disease conditions. In addition, Ninj1 integrates cellular adhesion and inflammatory signaling functions—processes that are crucial to the progression of CVD. The role of Ninj1 in maintaining cardiovascular physiological homeostasis as well as its involvement in disease pathogenesis has garnered extensive attention. Although current research reports on Ninj1 in the field of CVD remain relatively limited, this review endeavors to systematically summarize the studies on Ninj1 in the cardiovascular context, and to assess its strengths and limitations, with the aim of providing relevant references for future research on CVDs and inflammation analysis. This review focuses on this molecule, highlighting and elucidating its key regulatory effects across various pathological processes.

## 2. Overview of Ninjurin 1

The Ninj1 gene contains a 152‐amino acid open reading frame encoding a 16‐kDa polypeptide. Ninj1 has two hydrophobic transmembrane domains (amino acids 72 to 100 and 118–139) and an N‐glycosylation site [7]. In addition, the homophilic adhesion domain of Ninj1 is located in a 12‐residue region between Pro^26^ and Asn^37^ in the extracellular NH 2‐terminal (ENT) domain and consists of a cluster of tryptophan and arginine residues. Through homophilic interaction, Ninj1 mediates the contact between macrophages and vascular ECs. Ninj1 is also able to mediate membrane disruption through oligomerization, a classic hallmark of apoptosis. It initially accumulates in two places and then drills a wedge into the cell membrane, forming larger holes and lesions as more Ninj1 protein attaches to the initially drilled wedge. In this way, the cell membrane is torn until the cell collapses completely. The extracellular α‐helix of Ninj1 is inserted into the plasma membrane, and subsequent oligomeric Ninj1 monomers form amphiphilic filaments and rupture the plasma membrane [8]. More and more studies have shown that Ninj1 can play an important role in the regulation of inflammation, endothelial function, cell apoptosis, and other aspects by regulating P53, PI3K/Akt, P21, and other factors and signaling pathways, which are closely related to the occurrence and development of CVDs [9–11].

## 3. Ninj1 and AS

AS is the main pathological basis of CVDs, and inflammation and EC dysfunction play a key role in its occurrence and development. A large number of studies have revealed the close relationship between the inflammatory network and AS, and Ninj1 AS, a new inflammatory regulator, has gradually become the focus of its role in AS. Furthermore, clinical studies have confirmed that its circulating levels can serve as a biomarker for the risk of atherosclerotic stroke [12]. Studies have shown that the expression of Ninj1 is up‐regulated in a variety of inflammatory cells involved in AS, such as macrophages, ECs, and neutrophils. The up‐regulated expression of Ninj1 can promote the secretion and release of inflammatory factors, such as TNF‐α and IL‐6 by inflammatory cells, and the expression of Ninj1 has a complementary relationship with related inflammatory responses. Oxidative stress and activation of NF‐kB inflammatory pathway can also up‐regulate the expression of Ninj1 and participate in the occurrence and development of AS through pro‐inflammatory effect and promoting inflammatory cell aggregation [13].

Jeon et al. [14] found that Ninj1 expression was significantly up‐regulated in atherosclerotic lesions in humans and mice. Specifically, Ninj1 expression was up‐regulated in vascular ECs and mononuclear macrophages in response to inflammatory stimulation. The up‐regulated Ninj1 can interact with other inflammatory factors, activate the nuclear factor‐κB (NF‐κB) signaling pathway, promote the release of tumor necrosis factor‐α (TNF‐α), interleukin‐6 (IL‐6), and other pro‐inflammatory cytokines, and further aggravate the inflammatory response. The aggravation of the inflammatory cascade directly leads to the damage of vascular ECs, which makes it easier for monocytes to adhere to and migrate to the subintima of the blood vessel and take up lipids to form foam cells, thus accelerating the formation and development of atherosclerotic plaques. Moreover, in addition to inflammatory regulation, Ninj1 affects AS progression through structural remodeling mechanisms: affecting endothelial permeability, disrupting vascular endothelial integrity, and creating conditions for lipid deposition and inflammatory cell infiltration. Ninj1 can mediate the transendothelial migration of myeloid cells through its extracellular adhesion domain, thereby aggravating the inflammatory response. Animal experiments further confirmed the pathogenic role of Ninj1: Ninj1 gene knockout mice significantly reduced the degree of AS, macrophage infiltration, and lipid deposition in plaques. This suggests that inhibition of Ninj1 expression may be a potential strategy for the prevention and treatment of AS. Ninj1 knockdown could induce the proliferation of macrophages by activating mitogen‐activated protein kinase (MAPK) and inhibiting phosphatidylinositol 3‐kinase (PI3K). PI3K/AKT signaling pathway up‐regulates the expression of pro‐inflammatory genes and the recruitment of inflammatory macrophages in the lesion site through macrophage‐mediated inflammation. In addition, matrix metalloproteinase 9 (MMP 9)–cleaved soluble fragment of macrophage Ninj1 (sNinj1) was found to have anti‐AS effect both in vitro and in vivo. Leveraging this property, the administration of a soluble Ninj1‐mimetic peptide in a mouse model of AS was found to significantly inhibit the inflammatory activation and migratory capacity of macrophages, thereby effectively delaying the progression of AS. This suggests that this mimetic peptide may hold potential application value in the clinical treatment of AS.

From the perspective of clinical transformation, the soluble form of Ninj1 in serum is significantly higher in patients with coronary artery disease (CAD) than in healthy patients. This sNinj1 exhibits an antiatherogenic effect and is able to reduce the expression of pro‐inflammatory genes, thereby reducing the transmembrane migration of monocytes. Chen Fang et al. [15] investigated the relationship between plasma Ninj1 levels and the severity of coronary artery stenosis in patients with CAD and found that plasma Ninj1 levels were increased in patients with CAD. Elevated plasma Ninj1 level is associated with the severity of CAD and coronary artery stenosis, and plasma Ninj1 has the potential to predict CAD. These findings suggest that Ninj1 plays an important role in the development of AS.

Shin et al. [16] demonstrated that Ninj1 can bind to the lipid fraction of lipopolysaccharide (LPS) to modulate LPS‐induced inflammation in an in vitro model. Instead of Ninj1 amino acids 26 to 37 (homophilic binding domain), the Ninj1 region bound by LPS is Ninj1 amino acids 81 to 100 (first transmembrane domain). Interfering with the binding region between Ninj1 and LPS can precisely modulate LPS‐induced inflammation without affecting the adhesion function of Ninj1. Subsequent gene knockdown experiments confirmed that down‐regulation of Ninj1 reduced LPS‐induced secretion of inflammatory markers, such as nitric oxide (NO) and TNF‐α in macrophages. In addition, it has been found that amlodipine can reduce the expression of NADPH oxidase and NF‐kB in ECs, improve mitochondrial‐dependent oxidative stress and endoplasmic reticulum stress, and reduce the expression of Ninj1, thereby reducing monocyte adhesion and improving inflammatory stress in ECs [17].

Sun et al. investigated the expression and function of Ninj1 in AS using a comprehensive analytical approach incorporating molecular, functional, and histological methods. By silencing the Ninj1 gene in ECs, they assessed its effect on the NF‐κB signaling pathway and CXCL‐8 expression in endothelial dysfunction induced by oxidized low‐density lipoprotein (ox‐LDL). In in vivo experiments, administration of the Ninj1 inhibitor mPN12 peptide to ApoE knockout mice was used to evaluate its impact on plaque formation and composition, ultimately demonstrating that endothelial Ninj1 promotes AS by activating the NF‐κB/CXCL‐8 signaling axis. Both in vivo and in vitro models further confirmed that inhibiting Ninj1 reduces atherosclerotic plaque formation and lipid deposition. Additionally, in vitro results indicated that inhibiting Ninj1 preserves endothelial function by enhancing cell survival. It was ultimately concluded that Ninj1 drives AS through CXCL‐8‐mediated NF‐κB activation. By promoting endothelial dysfunction and vascular inflammation, Ninj1 is a critical mediator with the potential to be targeted in early atherogenesis [18]. These results suggest that Ninj1 may promote the formation of ox‐LDL, apoptosis, and endothelial dysfunction by accelerating inflammation and adhesion between mononuclear macrophages and ECs, which leads to the transfer of monocytes from ECs to subintimal cells and transformation into macrophages. The uptake of ox‐LDL into foam cells by scavenger receptors plays an important role in the occurrence and development of AS, which may provide new ideas for the diagnosis and treatment of AS and its related diseases and pharmacological mechanisms.

Although Ninj1 has been confirmed to be associated with inflammatory diseases, its endothelial‐specific role in AS remains unclear. Moreover, most current studies lack endothelial‐specific immune markers and quantitative analysis of immune‐infiltrating cells, resulting in insufficient direct evidence for the mechanisms of EC activation. Although some experiments have employed rescue assays to reactivate relevant pathways, the corresponding restoration of EC apoptosis and adhesive function remains to be explored, which could also serve as a focus for future research.

## 4. Ninj1 and Atrial Fibrillation (AF)

According to the 2020 European Society of Cardiology (ESC) guidelines for the diagnosis and management of AF, the global prevalence of AF among adults ranges from 2% to 4% [19]. Furthermore, a study focusing on the Asia‐Pacific region indicates that approximately 80 million individuals had AF in 2023, with projections suggesting a continued rise in prevalence. China exhibits one of the highest burdens of AF within the region, both in terms of prevalence and absolute patient numbers. AF represents the most common clinical arrhythmia, with primary mechanisms including atrial structural abnormalities, abnormal atrial electrical activity, and multiple wavelet reentry. Under the influence of aberrant ectopic foci, the atria lose their normal rhythm, generating rapid and disorganized electrical activity. This leads to abnormal cardiac contraction and relaxation, resulting in impaired cardiac function, atrial blood stasis, slowed blood flow, and consequent thrombus formation [20]. Supporting Clinical Evidence from Epidemiology: Fang et al. investigated the correlation between plasma Ninj1 levels and AF. Their findings demonstrate elevated plasma Ninj1 levels in AF patients, which correlate with left atrial enlargement and an increased risk of thromboembolism. Furthermore, plasma Ninj1 concentration showed a positive correlation with the CHA_2_DS_2_‐VASc score (assessing thromboembolic risk in AF) and left atrial volume in AF patients. Plasma Ninj1 concentration demonstrated superior predictive capability for AF development and progression compared to other clinical parameters. Elevated plasma Ninj1 levels are also associated with increased AF severity and recurrence risk [21]. Ninj1 contributes to organ fibrosis by promoting macrophage activation and enhancing the release of profibrotic factors (e.g., TGF‐β1) and pro‐inflammatory cytokines (e.g., IL‐1β and TNF‐α). At the cellular level, Ninj1 inhibits the PI3K/Akt signaling pathway, leading to down‐regulation of the anti‐apoptotic protein Bcl‐2 and subsequent apoptosis. Consequently, measuring plasma Ninj1 levels could serve as a biomarker candidate for assessing prognosis and guiding therapeutic decision‐making in AF patients [22].

## 5. Ninj1 and Cardiomyopathy

Kny et al. [23] found that Ninj1 expression was significantly up‐regulated in the myocardial tissue of patients with left ventricular hypertrophy caused by aortic stenosis, while its expression was at a lower level in noncardiomyopathy patients. In addition to the 16 kDa Ninj1 (Ninj1‐16), they also detected a 24 kDa variant of Ninj1 (Ninj1‐24), which is mainly expressed during myocyte hypertrophy and whose high molecular weight is caused by N‐glycosylation. Ninj1‐16 may be involved in muscle cell migration and fusion, while Ninj1‐24 mainly performs more myocyte‐specific functions, such as mediating myoblast differentiation and muscle cell growth. They further showed that the same results were obtained in the mouse cardiac hypertrophy model and in the cell model: Arginine‐vasopressin (Arg)–induced cardiac hypertrophy was attenuated in Ninj1 knockout cardiomyocytes. The possible mechanism is that Ninj1 can mediate intercellular adhesion to regulate cardiomyocyte hypertrophy and differentiation, promote myocardial hypertrophy and cardiomyogenesis, and further participate in ventricular remodeling. In addition, the gene localization of Ninj1 and dilated cardiomyopathy both involve human chromosome 9q22, and Ninj1 may be the pathogenic gene of dilated cardiomyopathy, which is involved in the occurrence and development of pathological myocardium [24, 25]. Different molecular weight variants of Ninj1 may play an important role in heart failure by participating in ventricular remodeling, and the specific mechanism needs to be confirmed by a large number of studies, which may provide new insights and directions for ventricular remodeling. In the future, it may be possible to modulate Ninj1 activity through gene editing or small‐molecule inhibitors, which may alleviate myocardial remodeling.

## 6. Ninj1 is Associated With Vascular‐Related Diseases

### 6.1. Ninj1 and Diabetic Vasculopathy

The role of Ninj1 in metabolic cardiovascular complications is emerging. EC dysfunction is important in the progression of diabetic vascular disease. Laura Toma et al. have found from clinical data that high levels of plasma glucose fluctuate in CVD. To evaluate the effect of oscillating glucose (OG) on EC function and to explore the molecular mechanisms involved, they subjected cultured human EC to OG, constant HG, or physiological concentrations for 72 h and assessed markers of inflammation, markers of oxidative stress, and markers of transendothelial transporters. The results showed that OG increased the expression of Ninj1, monocyte chemotactic proteins MCP‐1, RAGE, TNFR1, and VAMP‐3 and promoted monocyte adhesion. OG induced inflammatory stress by up‐regulating Ninj1 and stimulating human EC transporter. These effects were caused by ROS production or NF‐kB activation. Ninj1 is an important player in the inflammatory process of human ECs induced by TNFα. Ninj1 silencing inhibits OG‐induced up‐regulation of caveolin‐1 and VAMP‐3 in EC, which increases inflammatory stress, ROS production, and NF‐kB activation and stimulates transendothelial transport [26]. Laura Toma et al. also evaluated the potential of ginger extract (GEx) to reverse hemogenic endothelial cell (HEC) dysfunction and investigated its mechanism. The results showed that GEx reduced monocyte chemoattractant protein 1, vascular cell adhesion molecule 1, and monocyte adhesion [27].

Wang et al. [28] further focused on the role of Ninj1 in high glucose (HG) microenvironment: They found that Ninj1 was highly expressed in ECs of type 2 diabetic mice and was elevated in human umbilical vein endothelial cells (HUVECs) cultured with HG. In addition, Ninj1 levels were up‐regulated in ECs from clinical specimens of diabetic patients compared with nondiabetic tissues, indicating a biological link between Ninj1 and endothelial pathophysiology in diabetic patients. Under HG conditions, functional blockade of Ninj1 promoted endothelial tube formation and phosphorylation of endothelial nitric oxide synthase (eNOS). Ninj1 neutralizing antibody can promote EC lumen formation and eNOS phosphorylation under HG environment and inhibit HG‐induced apoptosis. Blocking Ninj1 inhibited caspase‐3 activation and increased Bcl‐2/Bax ratio, thereby inhibiting HG‐induced apoptosis in HUVECs. HG induced ROS overproduction, p38 MAPK, and NF‐κB activation, and gene overexpression of VCAM‐1, ICAM‐1, MCP‐1, and IL‐6, which were ameliorated by Ninj1 blockade. PI3K/Akt signaling pathway was involved in the regulation of Bcl‐2 expression and eNOS phosphorylation after Ninj1 blockade. Ninj1 can promote the expression of pro‐inflammatory genes, such as ICAM‐1, VCAM‐1, IL‐6, and MCP‐1; regulate the p38‐MAPK and NF‐κB pathways, and the formation of reactive oxygen species under HG conditions. However, Ninj1 could aggravate EC apoptosis under HG conditions not only by enhancing the activation of inflammation‐related caspase‐3, but also by mediating the inhibition of PI3K/Akt signaling pathway and reducing the expression of Bcl‐2. They confirmed that targeted blockade of Ninj1 preserves endothelial function by promoting EC survival, thereby maintaining blood flow levels. The study validated Ninj1 as a potentially effective target molecule for preventing and treating diabetes‐induced endothelial dysfunction, and our research provides a novel potential feasible strategy for the prevention and treatment of diabetic peripheral vascular complications, offering certain value for clinical exploration and application. However, the specific mechanism by which Ninj1 mediates EC dysfunction under HG conditions has not been fully elucidated at the genetic level. Elucidating the detailed mechanism of Ninj1 action could facilitate further clinical translation; therefore, subsequent research should focus on deeper analysis of this aspect. Furthermore, Ninj1 plays an important regulatory role in macrophage‐mediated biological functions, whereas this experiment only conducted phenomenological observations in this regard. The study of Ninj1’s regulation of macrophage function requires further in‐depth investigation.

### 6.2. Ninj1 and Thoracic Aortic Dissection (TAD)

TAD is a fatal CVD with rapid progression, high mortality, poor prognosis, and increasing incidence. Endarteritis is characterized by the extension of the intimal flap anteriorly and posteriorly from the site of the intimal tear. Aortic remodeling involves changes in vascular structure and function and is a histological feature of vascular heart disease, showing regional and heterogeneous changes. Yixuan Sheng et al. identified Ninj1 as a potential target for anti‐inflammatory strategies in TAD using spatial transcriptomics. Studies have shown that Ninj1 is highly expressed in TAD. By using Ninj1‐shrna to knock down the expression of Ninj1, the average diameter of the thoracic aorta (TA), the incidence of thoracic aortic aneurysm (TAA)/TAD and mortality can be significantly reduced. In addition, it can significantly reduce the fibrosis level of the aortic wall, elastic fiber rupture, and degeneration, and reduce the infiltration of T cells and macrophages in the aorta. After the administration of Ninj1 neutralizing antibody, the average diameter of TA, the incidence of TAA/TAD, and mortality were significantly reduced, and the fibrosis level of the TAD model. Additionally, the level of fibrosis, elastic fiber fragmentation, and degeneration in the aortic wall of the TAD model were markedly alleviated, accompanied by reduced infiltration of T cells and macrophages in the aorta. These findings suggest that the Ninj1 neutralizing antibody exerts a therapeutic effect on TAD. This study employed Tomo‐seq technology to focus on genes highly expressed in the TA, identifying a novel role for Ninj1 in TAD inflammation. Targeting Ninj1 attenuated the progression of TAD, indicating that Ninj1 may serve as a potential therapeutic target for TAD. Collectively, these results suggest that Ninj1 is involved in inflammation and tissue remodeling and plays an important role in vascular EC formation. However, this study is limited to animal experiments and has not yet been fully validated in human clinical research [29].

## 7. Ninjurin 1 is Associated With Ferroptosis‐Associated CVD

During the process of cardiac ischemia–reperfusion, the body will produce a series of pathological mechanisms that may lead to cell dysfunction and cell necrosis, including the production of oxygen free radicals and pro‐inflammatory gene products, Ca^2+^ overload, and energy disorders. In recent years, it has been found that a variety of nonapoptotic programmed cell death mechanisms are involved in the process of organ ischemia–reperfusion, including neutrophil extracellular traps, pyroptosis, and ferroptosis [30, 31]. Recent research by Ramos et al. [32] revealed that Ninj1 undergoes oligomerization during ferroptosis. Deficiency of Ninj1 protects macrophages and fibroblasts from ferroptosis‐associated PMR. Ninj1 mediates the release of cytosolic proteins and damage‐associated molecular patterns (DAMPs) from ferroptotic cells. Critically, Ninj1 serves as a key mediator of PMR and DAMP release not only in ferroptosis but also in PARP‐dependent cell death and H_2_O_2_‐induced necroptosis. While dispensable for initial ferroptotic events (e.g., lipid peroxidation, channel‐mediated calcium influx, and cell swelling), Ninj1 is essential for mediating the release of cytosolic proteins and DAMPs from ferroptotic cells. This suggests that targeting Ninj1 may represent a promising therapeutic strategy to mitigate ferroptosis‐associated inflammation. Furthermore, Ninj1 contributes to inflammasome‐driven pyroptosis. Following caspase‐dependent cleavage and activation of the executioner protein Gasdermin D (GSDMD), Ninj1 orchestrates downstream PMR. Although substantial ROS production occurs during pyroptosis, whether Ninj1 directly regulates ROS remains unclear. Additionally, Kayagaki et al. [33] demonstrated that Ninj1 antibody could effectively prevent Ninj1‐mediated cell membrane rupture and DAMPs release, which could reduce tissue damage and inflammatory response. This finding highlights the critical role of Ninj1 in regulating cell death and associated inflammation, and elucidates the mechanistic role of Ninj1 and provides experimental evidence supporting the use of Ninj1 inhibitors, which have demonstrated therapeutic potential. Although not all forms of ferroptosis (such as RSL3‐induced cell death) require the involvement of Ninj1, recent data indicate that this molecule plays a critical role in executing PMR during ferroptosis through Ninj1 oligomerization. Detailed regulatory mechanisms governing Ninj1 membrane organization and its oligomerization remain poorly understood, despite evidence implicating processes of membrane disk shedding and release. However, recent studies have shown that inhibiting Ninj1 oligomerization with monoclonal antibodies can block the final execution of other forms of regulated necrosis (such as necroptosis and pyroptosis) and at least partially suppress their immunogenicity, suggesting that targeting Ninj1 may represent a therapeutic option for reducing ferroptosis‐associated inflammation [1, 3, 34].

## 8. Conclusions and Prospects

In summary, the role of Ninj1 in regulating inflammatory responses has been increasingly recognized. Epidemiological studies have linked elevated Ninj1 expression to vascular diseases, such as AS and TAD, suggesting that it plays a key role in the occurrence and development of these inflammatory diseases [35].

Ninj1 plays a key role in CVDs by regulating inflammation, vascular EC proliferation and apoptosis, macrophage activation, myocardial cell proliferation, and apoptosis, and has a certain application prospect. However, the research of Ninj1 in CVD is still in the early stage, and there are still many problems that need to be further explored: The cellular and molecular mechanisms of Ninj1 in the occurrence and development of CVD need to be further studied, and the specific intervention methods and means of Ninj1 in CVD need to be further developed. In conclusion, Ninj1 has considerable potential in the diagnosis and treatment of CVDs. In the future, we can further study the mechanism of Ninj1, explore the therapeutic strategy of targeting Ninj1, carry out more clinical research, and strengthen multidisciplinary cooperation to provide a new theoretical basis and practical guidance for the prevention and treatment of CVD. To improve the outcomes of patients with CVD, it is believed that with the deepening of research, the mechanism and clinical application of Ninj1 in CVDs will be fully revealed, which will contribute to the diagnosis and treatment of CVD [9, 36, 37].

## Author Contributions

Conceptualization: Xiaohui Xu and Yuming Mu. Writing–original draft and review: Xiaohui Xu. revision and editing: Yuming Mu.

## Funding

This study was funded by the National Natural Science Foundation of China, 82560344; Excellent Talent and Innovation Team Cultivation Program of the First Affiliated Hospital of Xinjiang Medical University, cxtd202424.

## Disclosure

All authors read and approved the final manuscript.

## Conflicts of Interest

The authors declare no conflicts of interest.

## Data Availability

Data sharing is not applicable to this article as no datasets were generated or analyzed during this study.
